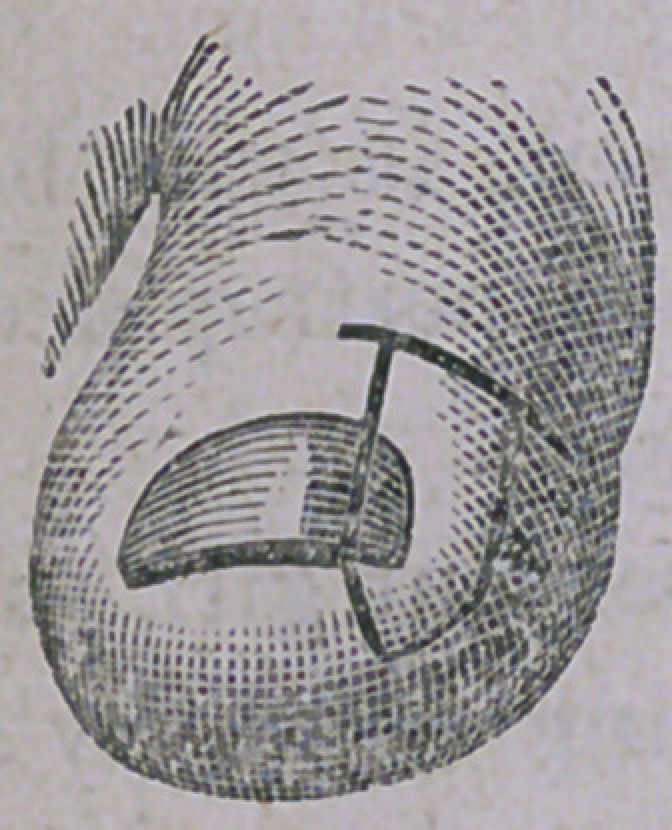# Ingrowing of the Toe-Nail

**Published:** 1874-05

**Authors:** Frank H. Hamilton


BUFFALO

Medical and Surgical Journal.

VOL XIII. MAY, 1874. No. 10

### Original Communications.

:o:

ART. I.—*Ingrowing of the Toe-Nail.* By Prof. FRANK H. HAMILTON, M. D.

The exceedingly troublesome affection, usually known as "ingrowing of the toe-nail," has been the subject of many ingenious surgical devices, most of which have been attended with partial or complete success in a large proportion of cases. Among the expedients familiar to Surgeons are, the removal of lateral pressure by the use of broad-toed shoes, cauterization of the sensitive granulations, lifting the margin of the nail with pledgets of lint, excision of the margin of the nail, scraping the nail in the direction of its length, so as to diminish the latteral growth and pressure.

Not one of these various expedients can, however, ensure to the patient complete and permanent relief. The fact is that in most if not in all of these cases the nail itself is not primarily at fault; the malady being caused by narrow shoes which crowd upon the sides of the great toe, and press one or both sides upwards, giving rise eventually to a hypertrophy of the tissues in this direction. There is in reality an up-growing of the flesh, and not an in-growing of the nail.

To accomplish absolute and permanent relief, then, it will be necessary to remove a part or the whole of the hypertrophied structure. This I have done in several ways, as follows.

First Method.—In June 1848, I operated upon a servant girl, assisted by Dr. John Trowbridge, of Buffalo, by cutting away, by a single incision the hypertrophied structure, and subsequently removing by incision and evulsion about one fourth of the nail on the same side. This was done to prevent the margin of the nail from interfering with cicatrization. The result was a complete success. but the cicatrization was slow; and in my next operation, July 10th, 1849, the case of Mrs. Pickard, aet. 25, I removed a portion of the margin of the nail by the same incision which cut away the hypertrophied tissue, cutting well back so as to include the root of the nail also. This also, healed slowly, but resulted in a perfect cure.

My notes contain other examples in which I have adopted the same practice and with the same results.

Second Method.—I am informed that Emmert, (German,) has cut away the excess of flesh, without including any portion of the nail. This operation I have myself repeated several times, and with satisfactory results, except that there has been delay in the cicatrization of the large open wound.

The delay in cicatrization, which ever of these methods is adopted, has led me at length to adopt a new method.

Third Method.—Nov., 19th, 1860, assisted by Dr. Squibb and Marvin, of Brooklyn, I operated upon Miss H., aet., 15. The inside of both great toes were affected in a similar manner, and she was, in consequence, scarcely able to walk. Having placed her under the influence of ether, I made first an incision transversely on the inner and dorsal aspect of the toe, about three lines back of the root of the nail, cutting to the bone. A second incision was made on the inner side of the toe, including the hypertrophied tissue, commencing about half an inch from the inner extremity of the first incision and terminating at the end of the toe. A third incision was then made, commencing from the transverse incision and terminating in the second incision at the end of the toe, including about one-fourth of the inner margin of the nail. These latter incisions, also extended to the bone. The intercepted tissues were then cut away. The matrix of a portion of the nail, both at its root and beneath the nail, were effectually extirpated, so that this position of the nail could not be reproduced. I was now able to *slide* up the remaining soft parts on the inner side of the toe, and, with a roller, to close the wound completely. The same operation was repeated upon the opposite foot On the 8th of December following both feet well.

This operation I have now repeated several times, and in all cases the cicatrization has been rapid and the cures complete.

## Figures and Tables

**Figure f1:**